# Understanding the Biomineralization Role of Magnetite-Interacting Components (MICs) From Magnetotactic Bacteria

**DOI:** 10.3389/fmicb.2018.02480

**Published:** 2018-10-23

**Authors:** Hila Nudelman, Yi-Zong Lee, Yi-Lin Hung, Sofiya Kolusheva, Alexander Upcher, Yi-Chen Chen, Jih-Ying Chen, Shih-Che Sue, Raz Zarivach

**Affiliations:** ^1^Department of Life Sciences and the National Institute for Biotechnology in the Negev, Ben-Gurion University of the Negev, Beer Sheva, Israel; ^2^Institute of Bioinformatics and Structural Biology, National Tsing Hua University, Hsinchu, Taiwan; ^3^Instrumentation Center, National Tsing Hua University, Hsinchu, Taiwan; ^4^Ilse Katz Institute for Nanoscale Science & Technology, Ben-Gurion University of the Negev, Beer Sheva, Israel

**Keywords:** biomineralization, magnetite-associated proteins, magnetotactic bacteria, MamC, Mms6, Mms7, protein–mineral interactions

## Abstract

Biomineralization is a process that takes place in all domains of life and which usually helps organisms to harden soft tissues by creating inorganic structures that facilitate their biological functions. It was shown that biominerals are under tight biological control via proteins that are involved in nucleation initiation and/or which act as structural skeletons. Magnetotactic bacteria (MTB) use iron biomineralization to create nano-magnetic particles in a specialized organelle, the magnetosome, to align to the geomagnetic field. A specific set of magnetite-associated proteins (MAPs) is involved in regulating magnetite nucleation, size, and shape. These MAPs are all predicted to contain specific 17–22 residue-long sequences involved in magnetite formation. To understand the mechanism of magnetite formation, we focused on three different MAPs, MamC, Mms6 and Mms7, and studied the predicted iron-binding sequences. Using nuclear magnetic resonance (NMR), we differentiated the recognition mode of each MAP based on ion specificity, affinity, and binding residues. The significance of critical residues in each peptide was evaluated by mutation followed by an iron co-precipitation assay. Among the peptides, MamC showed weak ion binding but created the most significant effect in enhancing magnetite particle size, indicating the potency in controlling magnetite particle shape and size. Alternatively, Mms6 and Mms7 had strong binding affinities but less effect in modulating magnetite particle size, representing their major role potentially in initiating nucleation by increasing local metal concentration. Overall, our results explain how different MAPs affect magnetite synthesis, interact with Fe^2+^ ions and which residues are important for the MAPs functions.

## Introduction

Many organisms across evolution rely on minerals of various shapes and sizes for a wide range of functions, including physical support, protection from external agents and navigation (Lijun et al., 2013). The process of mineral incorporation into biological systems is called biomineralization. Biomineralization involves the uptake of ions from the environment and their modulation into highly ordered structures, events that are subject to strict biology control (Mann, 2002). To create higher order inorganic structures, a solid phase organic matrix comprising polysaccharides, phospholipids, and mostly proteins is required (Addadi and Weiner, 1985). These proteins are involved in the three major steps of mineral formation, nucleation, crystal growth, and mineral size and shape. Most of the proteins from the organic matrix are intrinsically disordered proteins (IDPs), enriched in negatively charged residues, such as aspartate and glutamate, and undergo considerable post-translation modifications (Dunker et al., 2001; Weiner and Addadi, 2011; Kalmar et al., 2012).

In contrast to how minerals form spontaneously in nature, where extreme conditions (e.g., temperature, pressure, time, etc.) may be required, organisms provide an isolated environment in which a particular biological system cooperates with specific membranous pumps to create a saturating level for a specific ion (Weiner, 2003). The organic matrix plays several roles during mineral formation. First, the negatively charged residues attract and concentrate positive ions from the mineral solution. This leads to ion saturation at specific points and the start of mineral nucleation. Mineral nucleation requires a decrease in the free energy and critical size of seed particles in solution. Accordingly, the organic matrix lowers the free energy and stabilizes ions in solution to create a solid state with a stable minimal particle size that will grow into a stable crystal. Below this critical size, the mineral will dissolve. In addition to nucleation control, the organic matrix is involved in crystal growth and defining particle size and shape. Previous studies have shown that different proteins can directly interact with the mineral surface to direct mineral growth (Lowenstam and Weiner, 1989; Hunter, 1996; Mann, 2002).

Magnetotactic bacteria (MTB) synthesize a unique organelle, the magnetosome, that contains nano-magnetic particles of magnetite (Fe_3_O_4_) or greigite (Fe_3_S_4_) and thus represent an excellent model system for understanding biomineral formation (Lower and Bazylinski, 2013). The magnetosome is assembled into a chain or chains of well-defined nano-magnetic particles that are surrounded by a bilayer membrane and aligned according to the cell axis (Yamamoto et al., 2010). The magnetosome provides the bacteria with the ability to align to the Earth’s magnetic field so as to find an environment with appropriate oxygen levels (Katzmann et al., 2013). Magnetosome formation is under strict biological control and involves a specific set of genes organized into a specific gene cluster called the magnetosome island (MAI) (Grünberg et al., 2004).

Magnetosome island genes encode proteins with various functions which act during the three main stages of magnetosome synthesis. In the first two stages, magnetosome vesicles form followed by increased concentration of iron ions (Bazylinski and Frankel, 2004). The third stage in magnetosome synthesis starts with magnetite nucleation, followed by crystal growth, which is controlled by a specific set of magnetite-associated proteins (MAPs) (Hsu and Chan, 2011; Nudelman and Zarivach, 2014). These MAPs are all small acidic integral membrane proteins containing one to three transmembrane helices with specific motifs that can interact with iron ions and the magnetite surface, termed magnetite-interacting components (MICs) (Arakaki et al., 2014; Nudelman and Zarivach, 2014; Nudelman et al., 2016). These MICs are characterized by a high number of the acidic amino acids (aspartic acid or glutamic acid) and are known to be important for the biomineralization process.

One of the most studied and abundant MAPs in the magnetosome membrane (MM) is Mms6 (Amemiya et al., 2007; Rawlings et al., 2016; Raschdorf et al., 2017). According to secondary structure prediction, Mms6 contains a single transmembrane helix that includes a long C-terminal MIC oriented toward the magnetosome lumen. Previous studies showed that deletion of *mms6* caused defects in magnetite particle size and shape *in vivo* (Tanaka et al., 2011). Moreover, *in vitro* analysis of Mms6-MIC showed its ability to interact with magnetite particles and affect their size and shape while attached to a scaffold protein (Nudelman et al., 2016). Using nuclear magnetic resonance (NMR), it was recently shown that the Mms6-MIC can interact with Fe^2+^ to promote *in vitro* magnetite formation (Rawlings et al., 2016). A second abundant MAP in the MM is MamC (also known as Mms13 or Mam12) (Raschdorf et al., 2017). MamC is predicted to present a fold comprising two transmembrane helices separated by a MIC that points into the magnetosome lumen (Nudelman and Zarivach, 2014). Indeed, we previously determined the structure of the MamC-MIC while attached to a scaffold protein and showed it to adopt a helical structure. The helical structure of the MamC-MIC was shown to be important for its function during magnetite formation. Similar to the Mms6-MIC, *in vitro* analysis showed that the MamC-MIC affect the size and shape of magnetite particles. Further analysis showed that the helical structure of the MamC-MIC presents two negatively charged residues, Asp 66 and Glu 70, which contribute to the negatively charged protein surface (Nudelman et al., 2016). Mutating Asp 66 and Glu 70 to alanine abolished the ability of the MamC-MIC to interact with magnetite particles and affected their size and shape during an *in vitro* iron co-precipitation assay.

Other MAPs important for magnetite formation in the MTB *Magnetospirillum* have been identified, including Mms7 (MamD) and Mms5 (Scheffel et al., 2008). Like Mms6 and MamC, each of these proteins contains a MIC that is predicted to be directed into the magnetosome lumen. In each of the protein-MIC sequences listed in Supplementary Table S1, one finds a high number of negatively charged residues (Arakaki et al., 2003; Nudelman and Zarivach, 2014).

To better understand the role of MICs during magnetite formation, we considered three different MICs from MamC, Mms6, and Mms7. We performed biophysical characterization using NMR in the presence of different metal ions to obtain information on residues that may contribute to a metal ion-binding site. Furthermore, iron co-precipitation assays showed the effect of the different MIC residues and their mutants on magnetite formation. Each magnetite sample was characterized by X-ray diffraction (XRD) and electron spin resonance (ESR) to define differences between MamC-, Mms6- and Mms7-MIC functions. Our findings shed light on the functions of the three different MICs involved in magnetite biomineralization. The Mms6- and Mms7-MIC were shown to be mostly involved in ion binding, while the MamC-MIC has a larger effect on magnetite size and shape. Finally, we identified functional residues in each MIC.

## Materials and Methods

### Isotope-Labeling Peptide Preparation

To prepare MamC-, Mms6- and Mms7-MIC for NMR, the recombinant plasmids pET11b-intein-MamC, pET11b-intein-Mms6, and pET11b-intein-Mms7, respectively containing the MamC-, Mms6- and Mms7-MIC sequences, were constructed in the pET11b expression vector. *Escherichia coli* strain BL21 was used to express the recombinant fusion proteins. We grew the cultures in 500 mL of M9 medium with 100 μg mL^−1^ ampicillin at 37°C. The inducer IPTG was added when OD_600_ reached 0.6, at which point culture growth continued at 16°C overnight. The cells were harvested by centrifugation (4°C, 6000 × *g*) and lyzed in binding buffer (50 mM sodium phosphate, 300 mM NaCl, pH 7.0) using a homogenizer (GIM-03A, GW Tech., Taiwan). The soluble fraction was isolated by high-speed centrifugation (4°C, 36000 × *g*, 20 min). The over-expressed proteins were isolated from the supernatant using Ni-NTA resin (IMAC sepharose, GE Healthcare Life Sciences). The proteins were eluted with elution buffer (50 mM sodium phosphate, 300 mM NaCl, 400 mM imidazole, pH 7.0) and concentrated (Amicon Ultra centrifugal filter). Peptides self-cleaved from the coded intein in optimized buffer conditions and the peptide and intein were separated by High-Performance Liquid Chromatography (Shimadzu) with a C18 column and lyophilized. The uniformly ^13^C- and ^15^N-labeled peptide were prepared using an M9 medium with ^13^C-glucose and ^15^NH_4_Cl as supplements. The lyophilized peptide powders were dissolved in NMR buffer (25 mM MES, 10% D_2_O, pH 5.6).

### NMR Measurement and Resonance Assignment

All NMR data were acquired at 298 K on 600 and 850 MHz Bruker AVANCEIII spectrometers. The sequential backbone resonance assignments of H_N_, N_H_, C_α_, C_β_, C_o_ were carried out by [^1^H,^15^N]-HSQC, [^1^H,^13^C]-HSQC, HNCA, HN(CO)CA, HN(CA)CB, HN(CO)CACB, HNCO and HN(CA)CO. Spectra were generally acquired using a 15% non-uniformly sampled (NUS) protocol and reconstructed by packages hmsIST and NMRpipe (Delaglio et al., 1995; Hyberts et al., 2012).

### Metal Ion Titration in NMR Experiments

All titrated experiments were performed by simultaneous HSQC for simultaneously acquiring [^1^H,^15^N]-HSQC and [^1^H,^13^C]-HSQC. To prepare 50 mM Ni^2+^ and 10 mM Fe^2+^ stocks, we combined NiSO_4_ and FeO powders with NMR buffer and titrated the stocks into the peptide solutions. The final molar ratios of peptide to metal ions were 1:0.1, 1:0.2, 1:0.3, 1:0.5, 1:1.0, 1:1.5 and 1:2.0 for Fe^2+^ and 1:0.5, 1:1.0, 1:1.5, 1:2.0, 1:3.0, 1:4.0, 1:5.0, 1:7.0 and 1:10 for Ni^2+^. The NMR spectra obtained were processed by using the package NMRpipe and analyzed by the package of Sparky (Delaglio et al., 1995; Lee et al., 2015). The intensity ratios were calculated from the original HSQC peak intensities of free peptides and from the reduced intensities obtained in the presence of metal ions.

### Magnetite Co-precipitation Assay

All of the peptides were dissolved in Milli-Q-purified water. A solution of Fe_3_Cl and Fe_2_Cl, at a ratio of 2:1, was continuously sparged with N_2_, stirred (300 rpm) and slowly titrated with 0.1 M NaOH (100 mL/h) at room temperature. At pH 5, the peptide was added into the iron solution to a final concentration of 100 μM to detect the effect of the peptide on crystal size and shape. In control samples, an equal amount of Mili-Q-purified water (400 μl) was added at pH 5 to eliminate the effect of the liquid that the peptides were dissolved in. In all samples, titration was stopped between pH 9 and 10. All samples were stored in a BD GasPak EZ container system (Becton Dickinson and Company, Franklin Lakes, NJ, United States).

### Transmission Electron Microscopy (TEM)

Magnetite particle sizes and morphologies were detected by TEM using a Tecnai T12 TWIN transmission electron microscope (FEI). The synthesized magnetite was dehydrated with ethanol and embedded in epoxy resin. Ultrathin sections (60–80 nm) were prepared using a microtome (Leica Ultracut UCT). Sections of each sample were placed on 300 mesh copper grids. Imaging and size distribution analysis was performed on more the 200 nanoparticles for each sample. Magnetite particle size was measured manually using ImageJ 1.50i software. Size distribution curves and statistical calculations were performed using Statistica 13 and Microsoft Excel. Analysis of variance (ANOVA) was performed firstly to analyze the differences between the mean sizes of each sample, 1–11 (Alpha = 0.05). Second, we identified the significant between individual groups by using Tukey HSD for unequal N in order to get the *P*-values (Supplementary Table S2). Electron spin resonance (ESR).

Nanoparticle solutions (20 μl) were placed in a 20 mm length, 1 mm ID glass capillary and ESR spectra were recorded on an EPR-mini X-band spectrometer (Spin Ltd., Russia) at room temperature. A 10 G modulation, 0.01 time constant and microwave power level was chosen at the subcritical value of 2 mW to reach the best signal-to-noise ratio. In parallel, a glass tube with the 2,2-diphenyl-1-picrylhydrazyl (DPPH) standard was set at a 45° angle to the nanoparticle sample. When measured together, the DPPH standard and the tested sample provided a reference point for each measurement.

### X-ray Diffraction (XRD)

Sample characterization was performed by the X-ray powder diffraction method. Data were collected on a panalytical empyrean powder diffractometer equipped with a Pixel position sensitive detector (PSD) and a graphite monochromator on the diffracted beam. Cu K_α_ radiation (aaa = 1.541 Å) was used at 40 kV and 30 mA. The usual Bragg-Brentano q/2q was employed. Q/2q scans were run for 15 min in a 2q range of 10–60° with a step equal to ∼0.026°.

## Results

### NMR Titration Studies of the Different MICs

Three MICs derived from MamC, Mms6, and Mms7 are specifically involved in magnetosome assembly (Supplementary Table S1). The MamC-MIC contains 21 residues corresponding to the loop between the two transmembrane helices. The 22 residue-long Mms6-MIC is derived from the C-terminal portion of Mms6, while the Mms7-MIC is derived from Mms7 C-terminus and contains 17 residues. We used NMR to identify critical residues of each MIC involved in the process of magnetite nucleation. In such NMR experiments, metal ions are usually paramagnetic or diamagnetic and cause intensity decreases or chemical shift perturbations. We thus screened four different metal ions, Fe^2+^, Fe^3+^, Ni^2+^, and Zn^2+^, to establish binding specificity (Supplementary Figures S1, S2). We evaluated effects on NMR 2D homonuclear TOCSY. Titration of Zn^2+^ into the three MIC solutions did not bring significant chemical shift perturbation, indicating less interaction between the peptides and Zn^2+^ ions (Supplementary Figure S1). Meanwhile, resonance intensities were less affected since Zn^2+^ is diamagnetic. For Fe^3+^, titration generally attenuated resonance intensities, because Fe^3+^ ions are paramagnetic. Fe^3+^ in solution enhanced T_2_ relaxation and resulted in line-broadening of the residues. However, we did not observe a specific site that particularly responded to the titration. This indicated an absence of specific binding between the MICs and Fe^3+^ (Supplementary Figure S1). Titrating Fe^2+^ and Ni^2+^ induced significant intensity attenuation since both ions are paramagnetic. Fe^2+^ and Ni^2+^ affected specific regions where greater responses to the titrations were detected (Supplementary Figure S2). The two ions may have the similar recognitions, although Fe^2+^ was more effective than Ni^2+^ in signal quenching. The results indicated that Fe^2+^ and Ni^2+^ both specifically bind the three MICs and that the effects of Fe^2+^ and Ni^2+^ were comparable. However, the traditional ^1^H-^1^H spectra provided less resolution, especially for a peptide with a random-coil property, for which H_N_ resonances are mainly distributed into the narrow range of 8.0–8.5 ppm. The TOCSY analysis was not accurate due to signal overlapping. Therefore, to elucidate the details, we prepared uniformly labeled [^13^C,^15^N]-MICs and employed heteronuclear NMR spectroscopy.

### NMR Titration Studies of [^13^C,^15^N]-MICs

**FIGURE 1:**
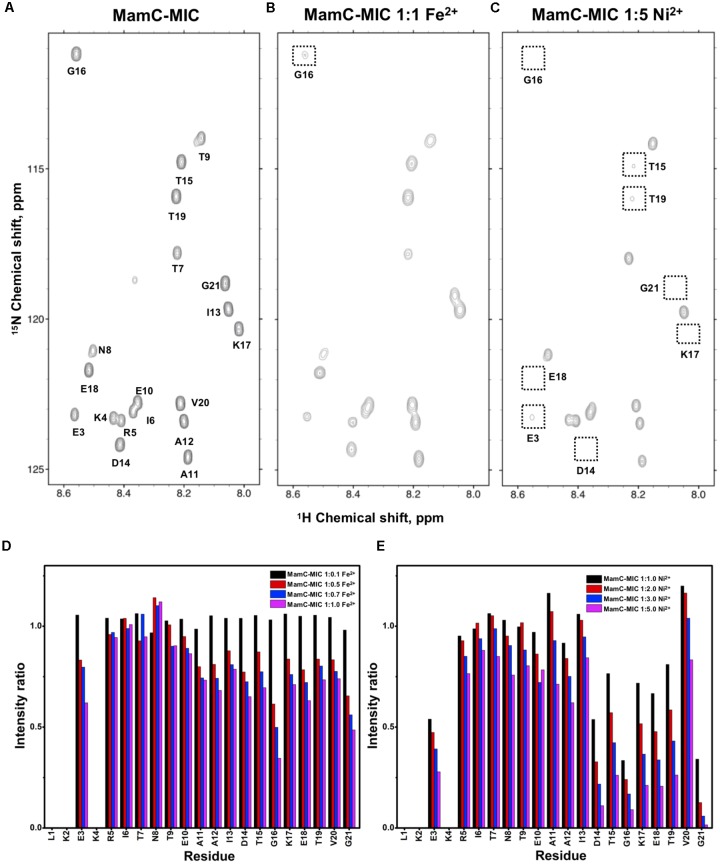
[^1^H,^15^N]-HSQC of the MamC-MIC in titration experiments. **(A)** The MamC-MIC in 25 mM MES buffer, pH 5.6. The metal ion was added to attain a molar ratio of **(B)** 1:1 for Fe^2+^ and **(C)** 1:5 for Ni^2+^, respectively. Intensity decay ratios in a series of titrations of **(D)** Fe^2+^ and **(E)** Ni^2+^, respectively.

**FIGURE 2:**
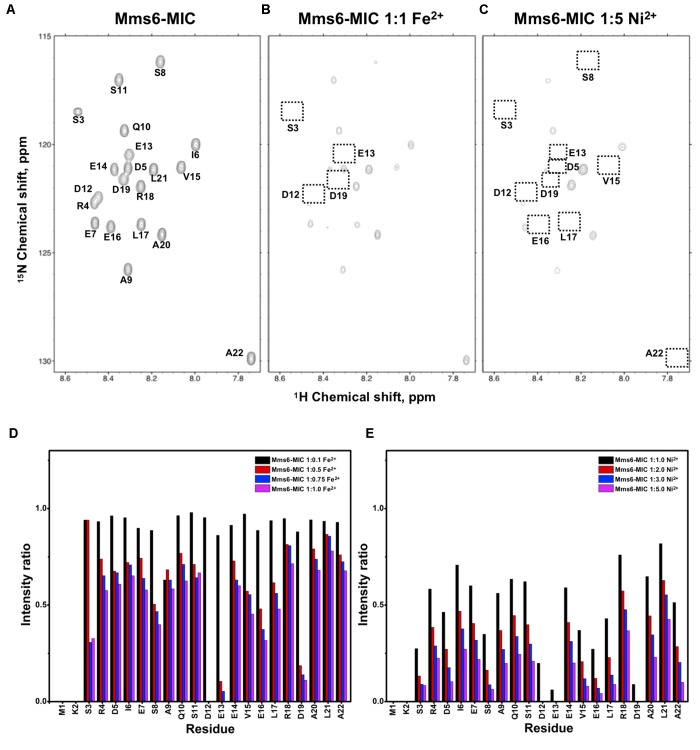
[^1^H,^15^N]-HSQC of the Mms6-MIC in titration experiments. **(A)** The Mms6-MIC in 25 mM MES buffer, pH 5.6. The metal ion was added to attain a molar ratio of **(B)** 1:1 for Fe^2+^ and **(C)** 1:5 Ni^2+^, respectively. Intensity decay ratio in a series of titrations of **(D)** Fe^2+^ and **(E)** Ni^2+^, respectively.

**FIGURE 3:**
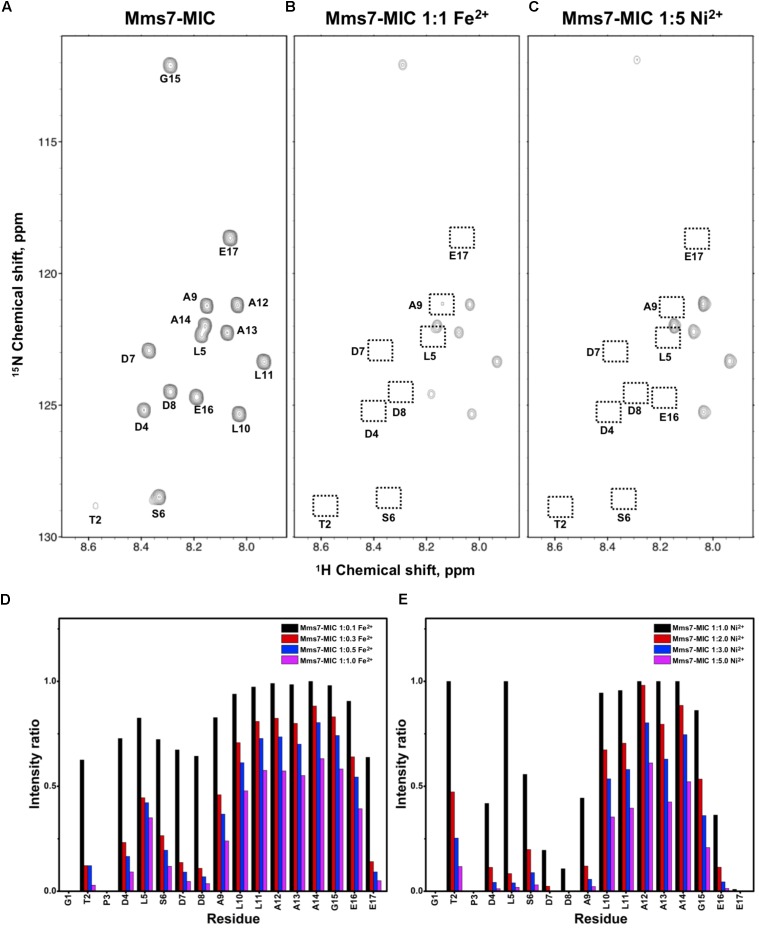
[^1^H,^15^N]-HSQC of the Mms7-MIC in the titration experiments. **(A)** Mms7-MIC in 25 mM MES buffer, pH 5.6. The metal ion was added to attain a molar ratio of **(B)** 1:1 for Fe^2+^ and **(C)** 1:5 Ni^2+^, respectively. Intensity decay ratio in a series of titrations of **(D)** Fe^2+^ and **(E)** Ni^2+^, respectively.

The former NMR titration experiments were performed using chemically synthesized peptides. To perform more advanced NMR studies, we prepared [^13^C,^15^N]-labeled MICs. We employed an *E. coli* system to express the peptides using ^15^N- and ^13^C-containing culture medium. Using an appropriate expression system in which the N-terminus of the MICs was linked to an intein, the MICs were separated upon intein C-terminal self-cleavage and the labeled MamC-/Mms6-/Mms7-MIC were purified. Heteronuclear NMR spectra providing satisfactory resolution were analyzed. The correlation of H_N_ and N on the [^1^H,^15^N]-HSQC spectra gave an excellent dispersion. We were thus able to fully assign all resonances, only missing the first two N-terminal residues due to fast solvent exchange (Figures 1A, 2A, 3A). When Fe^2+^ or Ni^2+^ ions were titrated into the three MIC solutions, reduced resonance intensities were obtained, although no chemical perturbation occurred. We quantified peak intensities to determine the reduction in ratio caused by the ions. Here, Fe^2+^ was generally more effective than Ni^2+^. Thus, two different titration ranges were used for the two ions. Specifically, the molar ratios of peptide to ion were 1:1 for Fe^2+^ and 1:5 for Ni^2+^.

In the case of the MamC-MIC, the effects derived in the presence of Fe^2+^ and Ni^2+^ were consistent (Figures 1B,C). The spectra of Ni^2+^ titration (at a ratio of 1:5) showed a higher effect in reducing H_N_-N resonance intensity than did Fe^2+^ (at a ratio of 1:1) (Figures 1D,E). We observed significant attenuation centered at Gly16, suggesting that the region containing Asp14-Thr19 could be the main site for binding. Of these residues, the negative charges of Asp14 and Glu18 might play roles in recruiting the ions. We also observed minor effects on other residues, such as Glu3, another acidic residue, and Gly21, the last residue in the C-terminus, presenting a negatively charged carboxylic group. Notably, binding between the MamC-MIC and Fe^2+^ or Ni^2+^ was not very strong. At the highest titration ratios tested, we still observed the most resonances, particularly in the case of Fe^2+^ at a 1:1 molar ratio (Figure 1B).

The Mms6-MIC had very significant intensity reduction effects in the titration experiments (Figure 2). Fe^2+^ specifically binds the Mms6-MIC, with major effects occurring at Asp12, Glu13, and Asp19, indicating the importance of these residues for binding (Figures 2B,D). Meanwhile, all residues had reduced intensities when 5-fold more Ni^2+^ ions were titrated (Figure 2C). The entire sequence was affected by Ni^2+^ addition, with Asp12, Glu13 and Asp19 being the most affected residues during the titration (Figure 2E). This could indicate the existence of two binding sites in the Mms6-MIC, corresponding to Asp12-Glu13 and Asp19, that cooperatively contribute to the significant binding. Meanwhile, we could conclude the importance of the region from Asp12 to Asp19, given that this region contains five negatively charged residues out of the eight comprising this span. We further noticed that Asp5 and Glu7, lying beyond this span, were less affected, implying the minor roles of these residues in ion-binding.

In Mms7-MIC titration experiments, the ions specifically affected the N-terminal portion of the peptide (Figure 3). The residues Thr2, Asp4, Leu5, Ser6, Asp7, and Asp8 showed severe intensity decreases. Upon Ni^2+^ titration, the N-terminal residues almost lost their intensities and only those of the C-terminal residues remained (Figures 3C,E). The Asp7-Asp8 pair may form the center of the binding site since the most significant effects occurred on the two residues. Furthermore, the C-terminal portion of the peptide contains poly-hydrophobic residues. It is not surprising that the hydrophobicity hampered the interaction with Fe^2+^ and Ni^2+^. In addition, we observed another significant effect that occurred at the last two residues, Glu16 and Glu17, possibly related to their acidic nature (Figures 3D,E). Overall, the Mms7- and Mms6-MIC behave as specific Fe^2+^/Ni^2+^-binding MICs.

### Secondary Structural Properties of the MamC/Mms6/Mms7-MIC

**FIGURE 4:**
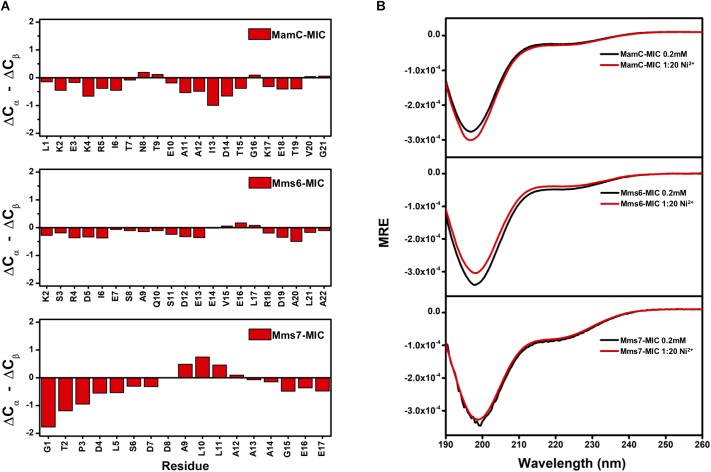
**(A)** Secondary structure prediction for MAP-MICs based on the NMR C_α_ and C_β_ chemical shift difference (ΔC_α_ – ΔC_β_). **(B)** CD spectra of MAP-MICs in the absence or presence of Ni^2+^.

We further evaluated whether MIC secondary structural properties were perturbed by ion binding. As observed in the TOCSY spectra, HSQC spectra also indicated the random-coil property of the free MICs, with H_N_ chemical shifts distributed in the 8.0–8.5 ppm range. These results were further confirmed by assessing the ^13^C_a_ and ^13^C_b_ chemical shifts. First, we collected ΔC_α_ and ΔC_β_ values that represent differences between the observed ^13^C chemical shifts and values obtained with random coils. The (ΔC_α_ – ΔC_β_) parameter was used to estimate secondary structural properties, whereby negative and positive values respectively represent α-helix and β-sheet structures (Wishart et al., 1992; Wishart and Sykes, 1994). The (ΔC_α_ – ΔC_β_) values were plotted as a function of residue number (Figure 4A). The relatively small values (<2 ppm) are indicative of no particular secondary structural tendency for any of the three MICs. The lack of secondary structure matched the measurements obtained by circular dichroism (CD) spectroscopy (Figure 4B). More importantly, the presence of Fe^2+^ and Ni^2+^ ions did not induce secondary structural properties, as we observed similar CD profiles during the titrations (Figure 4B). Although binding with Fe^2+^ and Ni^2+^ is specific, interactions were transient and not strong enough to induce any conformational changes.

### Binding Affinity Analysis

**FIGURE 5:**
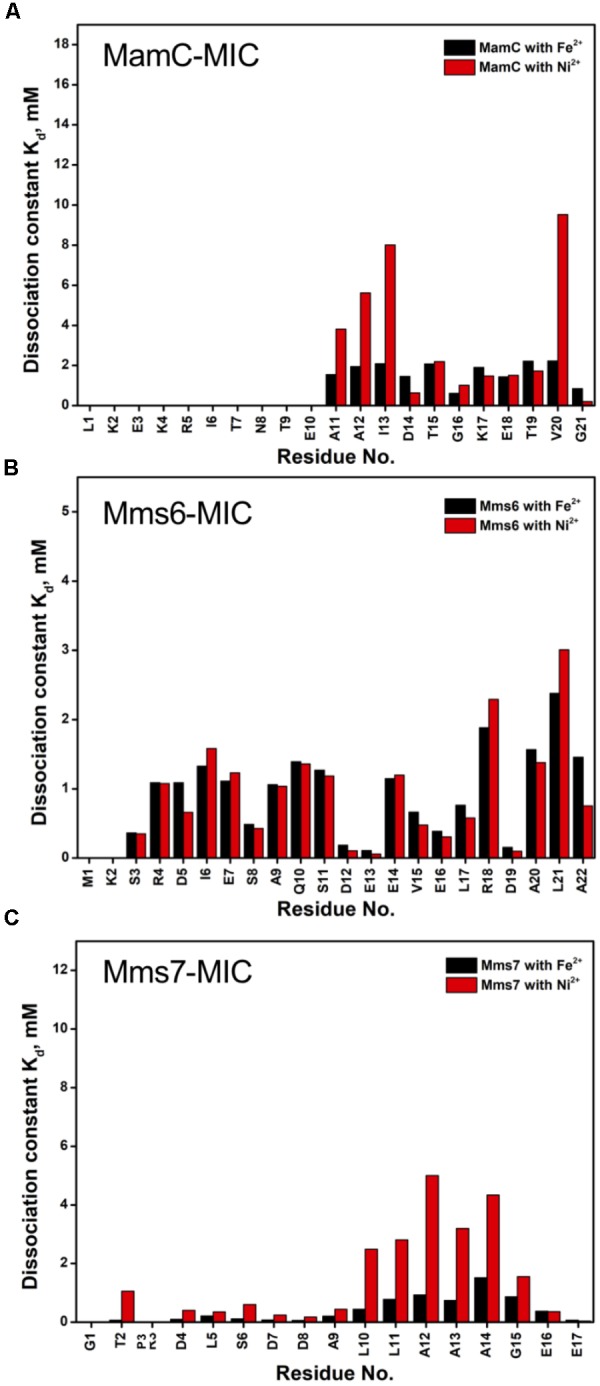
The dissociation constant K_d_ for each residue of MAP-MICs; MamC-MIC **(A)**, Mms6-MIC **(B)**, and Mms7-MIC **(C)**.

Based on the reduced ratios of resonance intensity, we determined the dissociation constant (K_d_) for each residue (Figure 5). In the MamC-MIC, stronger binding occurred at Asp14, Gly16 and Glu18 (Figure 5A), reflected in the low K_d_ values for these residues. Considering Asp14-Thr19 as comprising the binding region, we estimated averaged K_d_ values of 1.6 ± 0.6 mM for Fe^2+^ and 1.4 ± 0.5 mM for Ni^2+^, respectively. These K_d_ values are comparable, indicating similar binding affinities for the MamC-MIC binding of the two ions. Since the N-terminal portion induced much less intensity reducing, accurate estimation cannot be achieved and we excluded the analysis. For the Mms6-MIC, the fitted results revealed that the ions better bound Asp12, Glu13, Glu16 and Asp19 (Figure 5B). The results of Fe^2+^ and Ni^2+^ were similar. In considering these four residues alone, the K_d_ values are 0.21 mM ± 0.12 mM for Fe^2+^ and 0.14 ± 0.11 mM for Ni^2+^. These values are 10-fold stronger than those obtained for the MamC-MIC. The Mms6-MIC had a much higher ability for recruiting Fe^2+^ and Ni^2+^. The stronger affinity might be due to the existence of two ion-binding sites that mediate binding cooperatively. In the case of the Mms7-MIC, we defined the binding site as being located at the N-terminal portion, possibly corresponding to Asp4-Asp8. We obtained binding affinities for these residues of 0.11 mM ± 0.06 mM for Fe^2+^ and 0.36 ± 0.17 mM for Ni^2+^. The Mms7-MIC showed comparable Fe^2+^ binding affinity to that of the Mms6-MIC. Noticeably, most Mms7-MIC residues had higher K_d_ values for Ni^2+^ binding. This indicates that the Mms7-MIC had slightly weaker binding ability to Ni^2+^. The Mms7-MIC may possess selectivity in recognizing the two ions. Overall, the NMR results clearly indicated that ion binding occurred at Asp14 in the MamC-MIC, at Asp12, Glu13, and Asp19 in the Mms6-MIC and at Asp7 and Asp8 in the Mms7-MIC. Therefore, we designed further experiments to elucidate the roles of the individual residues in magnetic particle synthesis.

### *In vitro* Iron Co-precipitation Assay

**FIGURE 6:**
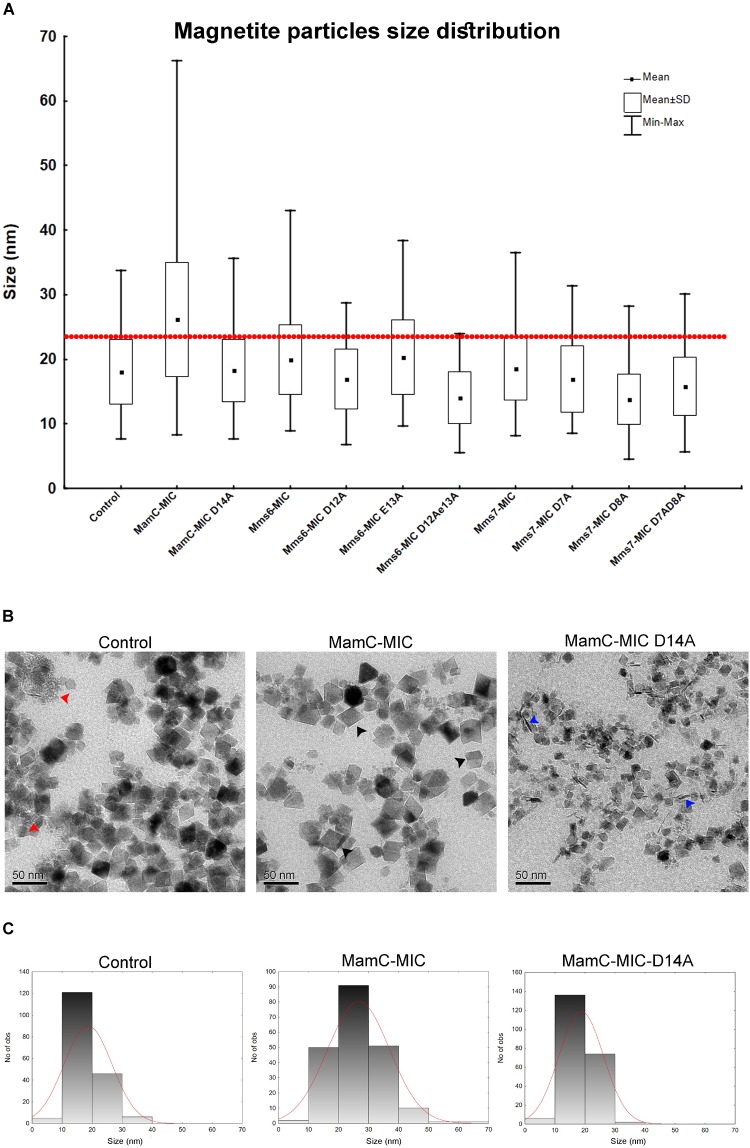
Magnetite particles size distribution from the iron co-precipitation assay results. **(A)** Size distribution statistical analysis summarizing all 10 samples with different peptides and a control sample. In all samples, the peptide concentration was 100 μM. The red line represents 90% of the control particle population. **(B)** TEM images of the control (MIC-free), MamC-MIC and MamC-MIC D14A samples. The red arrows point to primary particles which are less than 1 nm in size, black arrows represent cube-like-shaped particles and blue arrows represent rod-like-shaped particles. **(C)** Magnetite particles size 2D histogram for control, MamC-MIC and MamC-MIC D14A.

We employed an *in vitro* magnetite synthesis system to understand the roles of the MamC-, Mms6-, and Mms7-MIC in crystal nucleation and growth. Particle size distribution was determined in the presence or absence of the three different MICs in aqueous solution. Moreover, mutant MICs were generated on the basis of the NMR results to test the effects of specific residues on this assay. ANOVA test was performed between all the samples and reveal an *F*-value of 8.12e^+01^ and a *P*-value of 0, which point out on mean diversity between the different samples (Degree of freedom was 10). The ANOVA test was followed by Tukey HSD test which reveals the *p*-value between pairs of the different samples. Control experiments lacking any MIC revealed the presence of particles with an average size (AS) of 18 ± 0.37 nm, with 90% of the population being less than 24.6 nm (Figure 6A). The MamC-MIC had a significant effect in terms of modulating size distribution during the *in vitro* assay (Figure 6C). The presence of the MamC-MIC increased the AS to 26.1 ± 0.61 nm, together with widening size distribution to between 8.2 and 66.17 nm. The *P*-value between the control and the MamC-MIC sample was 1.49e^−05^, indicating significant differences between the samples, with the lower the *P*-value, the greater the statistical significance. The MamC-MIC D14A mutant led to the appearance of particles with an AS of 18.2 ± 0.32 nm and to a similar size distribution as seen with the control sample (*P*-value of 1.00e^+00^). In comparing the MamC-MIC and MamC-MIC D14A mutant, the *P*-value obtained (1.49e^−05^) is indicative of a major difference between the two.

In the case of the Mms6-MIC, the AS was slightly increased to 19.9 ± 0.36 nm, as compared to the control sample, yet with a *P*-value of 3.03e^−02^. Surprisingly, the Mms6-MIC E13A mutant did not have any major effect on magnetite size, which had an AS of 20.2 ± 0.38 nm, similar to that obtained with the wild type (WT) MIC (Supplementary Figure S3A). The *P*-value of 1.00e^+00^ between the Mms6-MIC and the Mms6-MIC E13A mutant may indicate a similar effect on particle size. It thus appears that Glu13 plays only a minor role in this aspect of crystal growth. Two other Mms6-MIC mutants related to Asp12, Mms6-MIC D12A, and D12A/E13A, produced particles with smaller AS values of 16.91 ± 0.32 nm and 14 ± 0.3 nm, respectively. These two samples showed major differences relative to the WT Mms6-MIC, based on the *P*-values measured (Supplementary Table S2).

The Mms7-MIC generated particles with an AS of 18.54 ± 0.29 nm, with 90% of the total population being less than 25.12 nm in size. According to the low *P*-value between the Mms7-MIC and the control sample (9.98e^−01^), a similarity exists between the two. Mms7-MIC mutants based on Asp7 and Asp8 all reduced particle average size. Noticeably, mutating Asp8 had a larger effect than did mutating Asp7, with the former leading to the appearance of smaller particles with an AS of 13.79 ± 0.27 nm (Figure 6A and Supplementary Figure S3B).

Generally, differences between all samples were mostly in the shape of the particles and the presence of primary particles, which are less than 1 nm in size. In the control sample, we could detect rounded particles together with those presenting an octahedral shape (Figure 6B). Moreover, we noticed primary particles in the control sample. In the MamC-MIC sample, the particles were more defined and only a minor number of primary particles were seen (Figure 6B).

Furthermore, differences in the shape and/or presence of primary particles were also seen with other samples. The variety in particle shape seen in the control samples was also seen in the MIC mutant samples, where a wide range of shapes was noted. In the MamC-MIC D14A sample, we detected rod-like shaped particles. These also appeared with the Mms7-MIC mutants. The particle shapes in the Mms6-MIC sample were less defined than those of MamC-MIC, while with the single mutant samples (i.e., Mms6-MIC D12A and Mms6-MIC E13A), we noticed more cube-like shaped particles (Supplementary Figure S3A). In Mms6-MIC D12AE13A sample, most of the particles were primary, with a small number of rod-like shapes. The Mms7-MIC sample mostly contained particles with a cube-like structure, while the mutants Mms7-MIC D7A, D8A and D7AD8A presented a diversity of shapes, such as cubic, rounded, rod and primary particles (Supplementary Figure S3B).

### XRD Analysis

**FIGURE 7:**
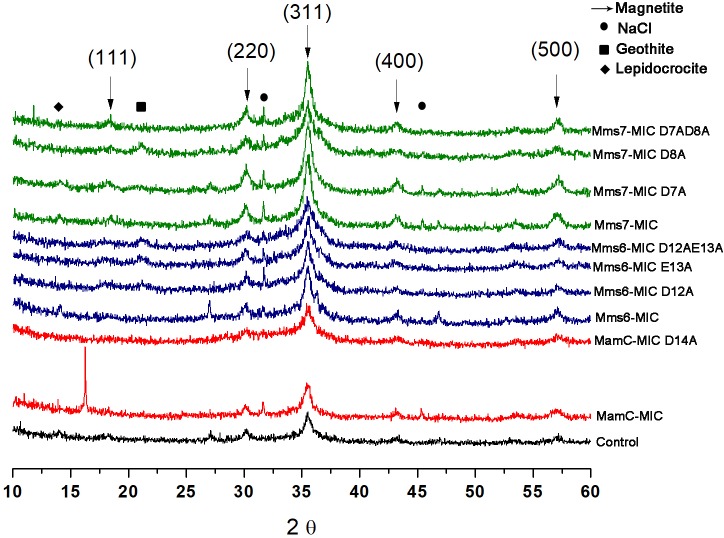
XRD for all the iron co-precipitation assay samples. Each MIC and its mutants are represented in different colors; MamC-MIC in red, Mms6-MIC in blue and Mms7-MIC in green. The control sample is represented in black. The arrows indicated the peaks that correspond to the magnetite XRD pattern and the different phases are in brackets.

XRD analysis was performed to assess crystal structure and component phases. All samples contained signals corresponding to the magnetite profile, including peaks at 18.269°, 35.422°, 30.095°, 43.052°, and 56.942°, which fit planes 111, 311, 220, 400, and 511, respectively (Figure 7). In the control and MamC-MIC samples, we also detected peaks which fit lepidocrocite (γ-Fe_3_O_2_), while goethite (α-Fe_3_O_2_) signals were detected in Mms6-MIC E13A, Mms6-MIC D12AE13A and Mms7-MIC D8A. In most of the samples, we could detect NaCl signals which may have formed while titrating 0.1 M NaOH into the iron solution. According to magnetite signal intensity, samples of Mms6-MIC, Mms6-MIC D12A and Mms7-MIC had lower intensity signals (i.e., not above the value of 3), as compared to the other samples (data not shown). For example, the strongest intensity signal of magnetite at 35.4° was higher than 30 in most of the samples.

### ESR Analysis

**FIGURE 8:**
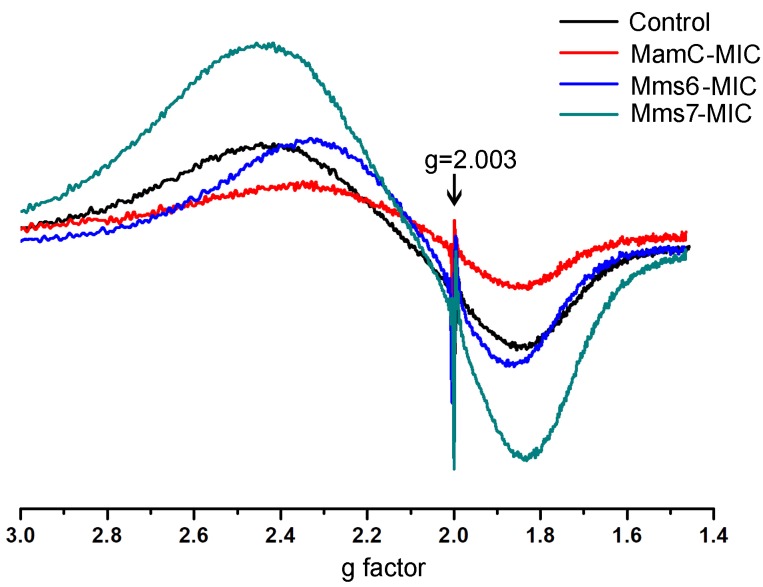
ESR spectra for the control, MamC-, Mms6-, and Mms7-MIC. The standard DPPH is marked with an arrow (g = 2.003).

ESR measurements of all of the magnetite samples were taken at room temperature and display a typical curve of the first derivative, with two symmetric peaks. Standard (DPPH) measurements were done in parallel to those of the magnetite samples. The standard presented the same curve in all samples and cutting point with the *x*-axis, with a g factor of 2.0036 (Figure 8). To calculate the g factor for each sample, we used the following equations:

$$g=\frac{\mathrm{hv}}{\beta B_{0}}$$

where *h* and *β* are constants (*h* = 6.626⋅10^−34^ J⋅s; *β* = 9.274⋅10^−28^ J⋅G^−1^), v represents the frequency used (9.45 MHz) during our measurements and B_0_ is the magnetic field.

All samples revealed two signals, with the first obtained in a low magnetic field with different g factor values and a second peak collected in a high magnetic field with similar g factor values (Supplementary Table S3). The ESR signal in all samples had the same curve shape with a single g factor value, reflecting the superparamagnetic properties of a sample, as shown in previous studies (Supplementary Figure S4) (Krzyminiewski et al., 2014; Dobosz et al., 2016). Differences in the sizes and shapes and the presence of peptides in the samples can affect the width and introduce some small variety in the *g* values measured. According to the iron co-precipitation assay and XRD patterns, differences exist mostly in the size and shape of the particles. Moreover, the presence of peptide in a sample and organic-non-organic interaction may influence the broad and symmetrical ESR signal (Sayar et al., 2006).

## Discussion and Conclusion

The NMR results presented here firstly revealed the differences in the binding of the four metal ions, Fe^2+^, Fe^3+^, Ni^2+^, and Zn^2+^, by the MICs. The MICs showed binding specificity for titrating Fe^2+^ and Ni^2+^ while titrating with Fe^3+^ and Zn^2+^ revealed less specificity and significant effects. The differences between Fe^2+^ and Fe^3+^ might be due to the different oxidation states which influence charge density and metal coordination. Similar to the recent publication of Rawlings et al. (2016), NMR measurements of the Mms6-MIC showed the same changes during Fe^2+^ titration. In contrast, our results showed less specific interaction with Fe^3+^ and no significant conformational changes during the titrations. Zn^2+^ had no effect on the signal during titration. The mechanism of ion-binding might resemble that reported in previous studies (Arakaki et al., 2003; Rawlings et al., 2016). Other than oxidation state differences, the dominant geometry of Zn^2+^ is tetrahedral, which is different from the octahedral geometry of Fe^2+^, Fe^3+^, and Ni^2+^ (Barber-Zucker et al., 2017). The similar geometry and oxidation states of Ni^2+^ and Fe^2+^ may explain the interactions of the same residues in the different MICs.

Individual MIC residues presented different NMR signal decays during titrations. Not surprisingly, the major intensity changes were distributed to the negatively charged residues. The MamC-MIC showed major signal changes at Asp14, a residue that was already known to play a role in magnetite nucleation and binding (Nudelman et al., 2016), as also reflected in the current iron co-precipitation assay. Noticeably, Gly16 was significantly affected although glycine is a non-polar residue theoretically not involved in ion-binding. The literature indicates that peptides comprising glycine hexamers moderately interact with magnetite nanoparticles (Schwaminger et al., 2017). This might imply a role for Gly16 in biomineralization. Meanwhile, the Mms6-MIC showed major responses at Asp12 and Glu13, as did the Mms7-MIC at Asp7 and Asp8. We did not see any preference of the metal ions in binding Asp or Glu. The two residues contributed less differences in recognizing Ni^2+^ and Fe^2+^. One result showed that when adjacent, the two acidic residues significantly enhanced the interaction with ions, as observed in the enhanced binding affinities of the Mms6- and Mms7-MIC, where the two peptides showed 10-fold higher binding affinity than did the MamC-MIC. In addition, the two ions did not bring about significant pseudo-contact chemical shifts (PCS) during the titrations. This indicates that both Ni^2+^ and Fe^2+^ had no particular orientation in binding the MICs.

We observed that the presence of the MamC-MIC promoted the assembly of larger magnetite particles in the iron co-precipitation assay, whereas those particles synthesized in the presence of the Mms6- or Mms7-MIC generally had similar sizes than obtained in the control experiment. Moreover, those particles promoted by the MamC-MIC contained a more defined shape with cube-like symmetry, as compared to those generated in the presence of the Mms6- or Mms7-MIC. It is surprising that the MamC-MIC showed the weakest binding of ions and, moreover, created the most significant effect in enhancing magnetite particle size. Instead, the strong ion binders Mms6- and Mms7-MIC had almost no effect in modulating magnetite particle size. This phenomenon indicates that the regulating magnetite particle formation and recruiting metal ion functions may be decoupled. The strong ion-binding affinity observed with Mms6 and Mms7 might represent a potent role in increasing local metal ion concentrations, an event critical for initial nucleation. MamC, although showing less ion-binding affinity, mainly contributes to modulating magnetite particle size and shape and potentially recognizes particles. The feature could be correlated with the orientation of the different MICs in the magnetosome lumen. The MamC-MIC represents a loop between two transmembrane helices and might contain a confined structure when bound to the magnetic particle. Instead, the Mms7- and Mms6-MIC are located at the C-terminal end, just next to a transmembrane helix. They thus represent very flexible sequences. The different orientations could contribute to the various effects seen during iron precipitation.

Together with their negative charges, most known proteins involved in biomineral synthesis assume a disordered structure and do not adopt any secondary conformation, such as α-helices or β-sheets (Kalmar et al., 2012; Boskey and Villarreal-Ramirez, 2016). Such structural flexibility may contribute to ion cluster arrangement followed by nucleation and mineral formation. In our previous study, we, however, showed that the MamC-MIC, while attached at its N-terminus to a scaffold protein, adopts a helical structure (Nudelman et al., 2016). Here, the MICs did not present any secondary structure, according to the NMR results. As only MICs were used in the titration experiments performed here, one of the reasons for the lack of secondary structure could be that in nature, the MamC-, Mms6- and Mms7-MIC are attached to the magnetosome membrane (Nudelman and Zarivach, 2014), where the individual proteins might interact with each other to form the clusters needed for function. Extensive research focusing on the entire proteins is thus required.

The particles formed in the present study revealed the typical signature of magnetite particles in XRD. Each peptide showed different signals during metal titration, with such changes accruing on negatively charged residues in each case. The Mms6-MIC sequence contains seven negatively charged residues in total but the three residues Asp12, Glu13 and Asp19 showed the most significant changes during iron titration. Similar to the *in vivo* analysis, a single mutation on Asp12 and Glue13 showed a significant effect on magnetite crystal shape and size in MTB (Yamagishi et al., 2016). One can thus assume that the other negative residues have some effect on ion binding and contribute to the negatively charged environment. Similarly, in the Mms7-MIC, which contains five negatively charged residues in total, only two, Asp7, and Asp8, showed the most significant changes during metal titrations. In contrast, the MamC-MIC contains only four negatively charged residues of which Asp14 showed the most noticeable signal changes during metal titration.

XRD analysis of samples from the iron co-precipitation assay indicated the major presence of magnetite. All samples presented ESR spectra with the same shape, corresponding to that of magnetite particles. Minor changes between the samples were mostly seen in terms of curve width, with a small shift in the g factor value (Supplementary Figure S4). These minor changes could result from the MICs being in the solution and/or differences in particle sizes.

In summary, our results shed light on the residues that are involved in magnetite formation and their abilities to interact with specific ions. According to the literature, Mms6 is involved in magnetite nucleation, with deletion of *mms6* or only its C-terminus causing changes in the size and shape of magnetite particles (Tanaka et al., 2011; Kashyap et al., 2014; Yamagishi et al., 2016). In this study, *in vitro* iron co-precipitation by the Mms6-MIC had a minor effect on magnetite size and shape, which indicates that the major role of the protein is not realized during crystal growth. It can be assumed that deletion of one of the MAP-encoding genes would initiate a chain reaction by interfering with the functions of other MAPs, ultimately leading to defects in particle size and shape (Yamagishi et al., 2016). Experiments with the Mms7-MIC revealed similar results, with only a minor effect on crystal size and shape being seen, which may also indicate that the major role of this protein is seen during crystal nucleation and ion binding and less so during crystal growth. In contrast, the MamC-MIC had the biggest effect during iron co-precipitation, possibly indicative of its function during magnetite-crystal growth. The ability to control magnetite formation can be useful for many industrial or medical applications which are currently using nanomagnetic particles (NPs) (Lang et al., 2007; Arakaki et al., 2008; Bao et al., 2015). Our results demonstrated the advantageof controlling magnetite size and shape by adding a specific peptide during synthesis (Li et al., 2016; Kudr et al., 2017). Moreover, those MICs can be used as an adaptor to interact with the NPs in one end, while on the other end it can be bound to a functional ligand (Arakaki et al., 2003; Nudelman et al., 2016). Our findings thus reinforce the assumption that each protein serves a different function during magnetite formation and the involvement of specific residues from each protein in this process. Understanding the specific function of residues or peptide sequences during the different stages in the biomineral formation (ions saturation, nucleation and crystal growth), can provide the ability to designing synthetic peptide which mimicking a desire biomineralization-function.

## Author Contributions

HN and Y-ZL contributed equally to this work. All authors contributed to the manuscript, read and approved the submitted version.

## Conflict of Interest Statement

The authors declare that the research was conducted in the absence of any commercial or financial relationships that could be construed as a potential conflict of interest.
