# Supplementary material for: Understanding the Biomineralization Role of Magnetite-Interacting Components (MICs) From Magnetotactic Bacteria
Source: Front Microbiol. 2018 Oct 23;9:2480. doi: 10.3389/fmicb.2018.02480 (PMC6206293; doi:10.3389/fmicb.2018.02480)
Supplement: Supplementary file 1 [file Data_Sheet_1.PDF]

Supplementary Material

Understanding the biomineralization role of magnetite-interacting components (MICs) from magnetotactic bacteria

Hila Nudelman[1#], Yi-Zong Lee[2,3#], Yi-Lin Hung[2,3], Sofiya Kolusheva[4], Alexander Upcher, Yi-Chen Chen[2], Jih-Ying Chen[2], Shih-Che Sue[2]* and Raz Zarivach [1,4]*

[1] Department of Life Sciences and the National Institute for Biotechnology in the Negev, Ben-Gurion University of the Negev, Beer Sheva, Israel

[2] Institute of Bioinformatics and Structural Biology, National Tsing Hua University, Hsinchu, Taiwan

[3] Instrumentation Center, National Tsing Hua University, Hsinchu, Taiwan

[4] Ilse Katz Institute for Nanoscale Science and Technology, Ben-Gurion University of the Negev, Beer Sheva, Israel

***Correspondence:**
Raz Zarivach
zarivach@bgu.ac.il
Shih-Che Sue
scsue@life.nthu.edu.tw

[#]Author contributed equally to this work.

1 Supplementary Figures and Tables

1.1 Supplementary Figures

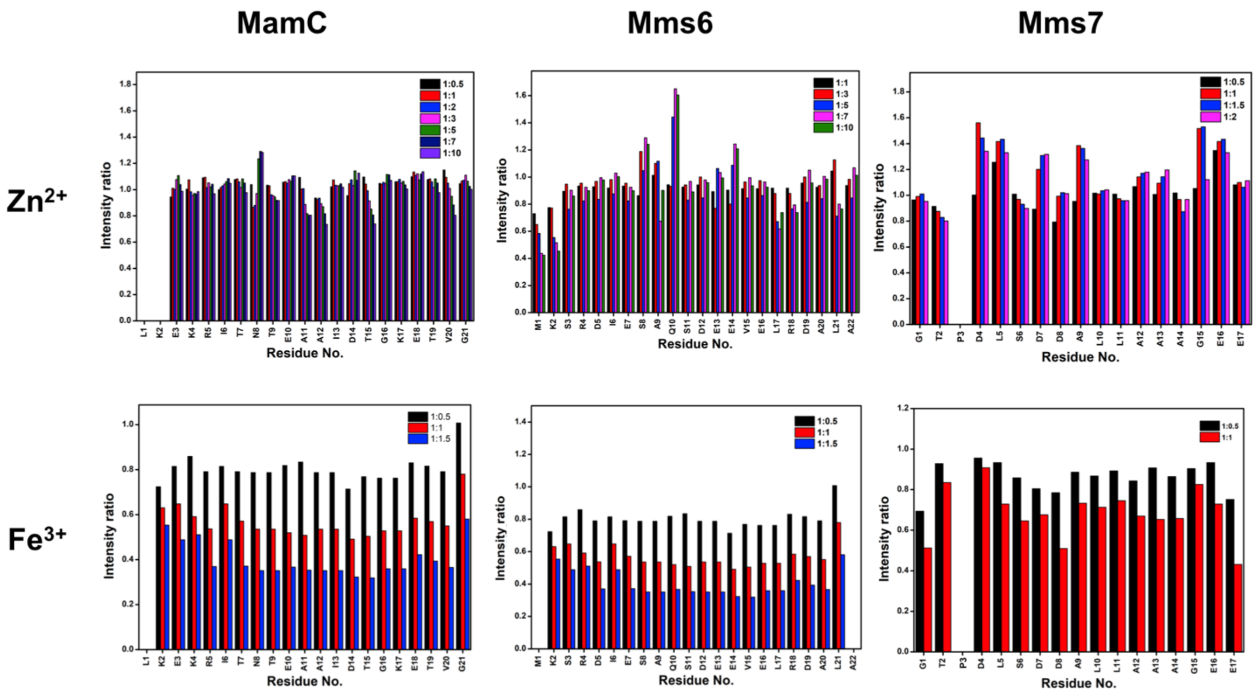

Supplementary Figure 1: NMR titration of Zn^{2+} and Fe^{3+} to MICs. The peaks intensity ratios are derived from H_N-H_A correlations measured from 2D homonuclear TOCSY.

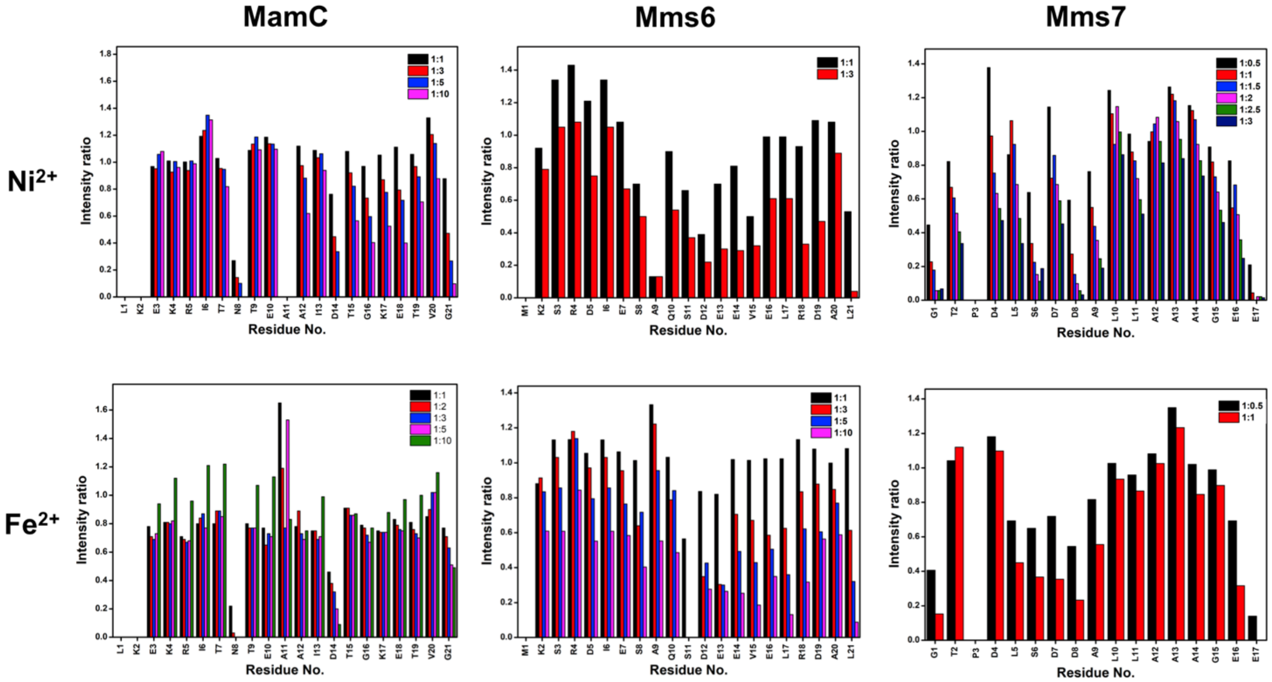

Supplementary Figure 2: NMR titration of Fe^{2+} and Ni^{2+} to MICs. The peaks intensity ratios are derived from H_N-H_A correlations measured from 2D homonuclear TOCSY.

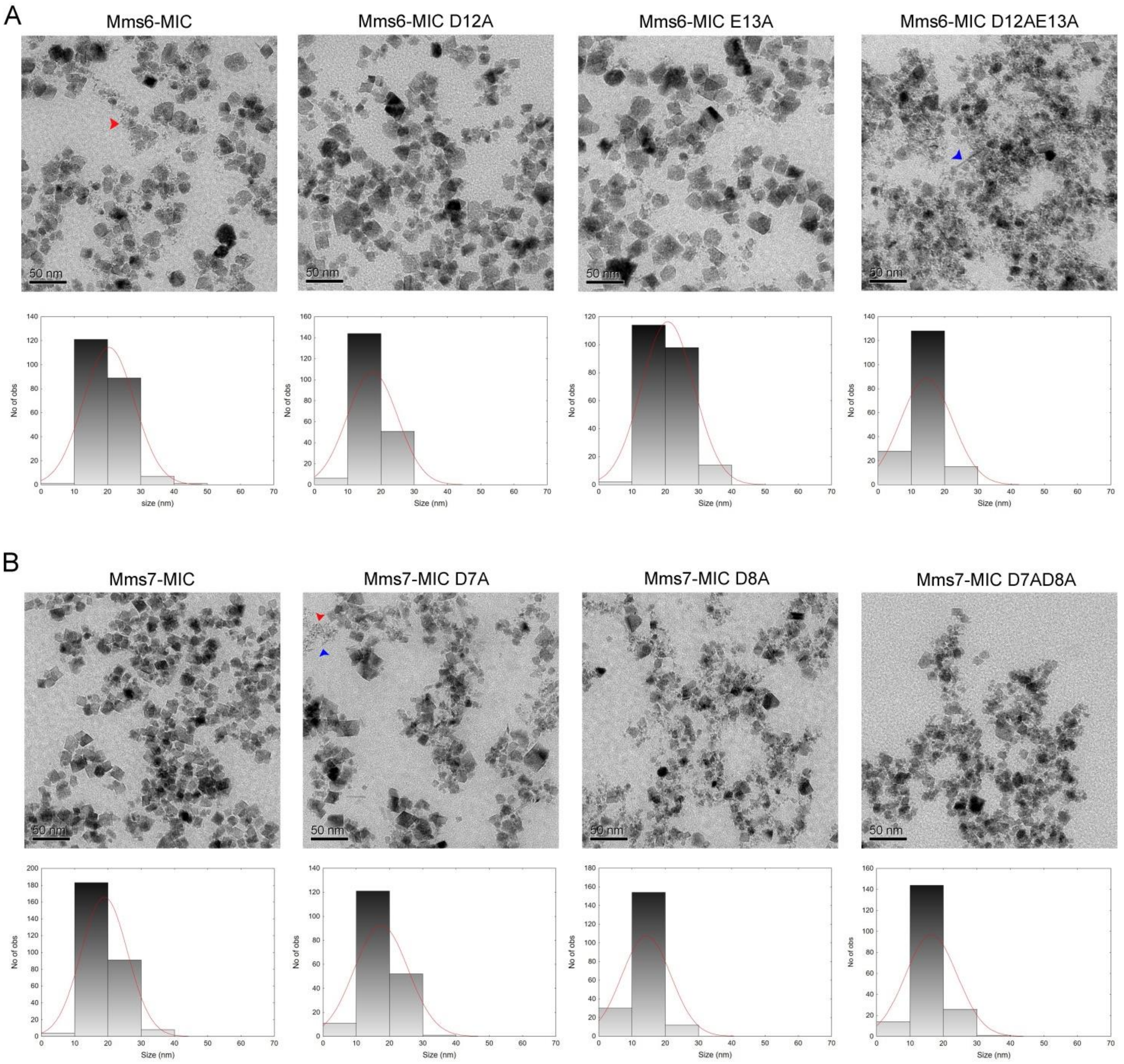

Supplementary Figure 3. Iron co-precipitation assay TEM images and 2D histograms for Mms6- and Mms7-MIC. (A) TEM images of Mms6-MIC and the mutated MIC samples. A 2D histogram presents the particle size distribution for each sample. (B) TEM images of Mms7-MIC sample and the mutated MIC samples. A 2D histogram presents the particle size distribution for each sample.

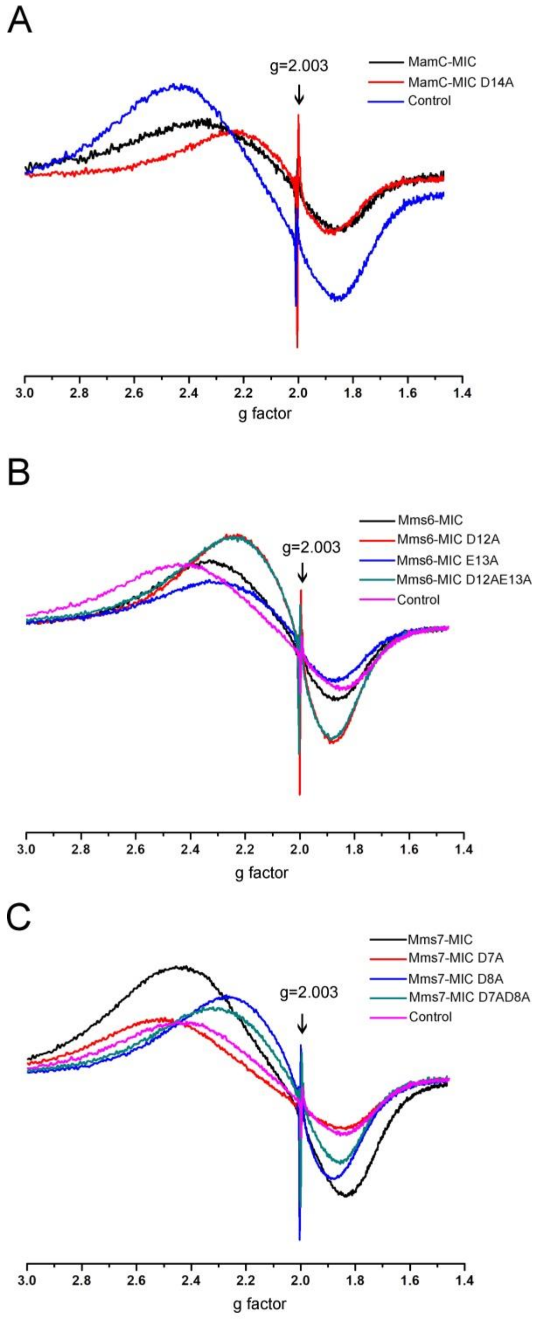

Supplementary Figure 4. ESR spectra for the mutant's samples and their WT-MICs. (A) Control, MamC-MIC and MamC-MIC D14A samples. (B) Control, Mms6-MIC, Mms6-MIC D12A, Mms6-MIC E13A and Mms6-MIC D12AE13A samples. (C) Control, Mms7-MIC, Mms7-MIC D7A, Mms7-MIC D8A and Mms7-MIC D7AD8A. The standard DPPH is marked with an arrow (g= 2.003).

1.2 Table supplementary

Supplementary Table 1. Summary of the different magnetite interacting components (MICs). Marked in red are the amino acids which are recognized by NMR to have a role during ion binding. All MICs are found in *M. magneticum* strain AMB-1.

Protein	Magnetite Interacting Component (MIC)-Sequence	MIC position in the full-length protein	A number of negatively charged a.a.	Length
MamC	[1]LKEKRITNTEAAIDTGKETVG[21]	Leu57-Gly77	4	**21**
Mms6	[1]MKSRDIESAQSDEEVELRDALA[22]	Met112-Ala133	7	**22**
Mms7	[1]GTPDLSDDALLAAAGEE[17]	Gly298-Glu314	5	**17**

Supplementary Table 2. Tukey HDS test results. P values between the Iron co-precipitation samples. The values which marked in red indicate on significant differences between the mean values of each sample ($P < 0.05$).

	Control	MamC-MIC	MamC-MIC D14A	Mms6-MIC	Mms6-MIC D12A	Mms6-MIC E13A	Mms6-MIC D12AE13A	Mms7-MIC	Mms7-MIC D7A	Mms7-MIC D8A	Mms7-MIC D7AD8A
**Control**		1.49e^{-05}	1.00e^{+00}	3.03e^{-02}	5.69e^{-01}	1.80e^{-03}	1.49e^{-05}	9.98e^{-01}	6.52e^{-01}	1.49e^{-05}	3.04e^{-03}
**MamC-MIC**	1.49e^{-05}		1.49e^{-05}	1.49e^{-05}	1.49e^{-05}	1.49e^{-05}	1.49e^{-05}	1.49e^{-05}	1.49e^{-05}	1.49e^{-05}	1.49e^{-05}
**MamC-MIC D14A**	1.00^{e+00}	1.49e^{-05}		5.66e^{-02}	2.50e^{-01}	3.42e^{-03}	1.49e^{-05}	1.00e^{+00}	3.26e^{-01}	1.49e^{-05}	2.58e^{-04}
**Mms6-MIC**	3.03e^{-02}	1.49e^{-05}	5.66e^{-02}		1.54e^{-05}	1.00e^{+00}	1.49e^{-05}	1.46e^{-01}	1.65e^{-05}	1.49e^{-05}	1.49e^{-05}
**Mms6-MIC D12A**	5.69e^{-01}	1.49e^{-05}	2.50e^{-01}	1.54e^{-05}		1.49e^{-05}	2.35e^{-05}	3.89e^{-02}	1.00e^{+00}	1.52e^{-05}	6.53e^{-01}
**Mms6-MIC E13A**	1.80e^{-03}	1.49e^{-05}	3.42e^{-03}	1.00e^{+00}	1.49e^{-05}		1.49e^{-05}	1.05e^{-02}	1.49e^{-05}	1.49e^{-05}	1.49e^{-05}
**Mms6-MIC D12AE13A**	1.49e^{-05}	1.49e^{-05}	1.49e^{-05}	1.49e^{-05}	2.35e^{-05}	1.49e^{-05}		1.49e^{-05}	2.49e-^{05}	1.00e^{+00}	5.21e^{-02}
**Mms7-MIC**	9.98e^{-01}	1.49e^{-05}	1.00e^{+00}	1.46e-01	3.89e^{-02}	1.05e^{-02}	1.49e^{-05}		6.38e^{-02}	1.49e^{-05}	1.85e^{-05}
**Mms7-MIC D7A**	6.52e^{-01}	1.49e^{-05}	3.26e^{-01}	1.65e^{-05}	1.00e^{+00}	1.49e^{-05}	2.49e^{-05}	6.38e^{-02}		1.53e^{-05}	6.29e^{-01}
**Mms7-MIC D8A**	1.49e^{-05}	1.49e^{-05}	1.49e^{-05}	1.49e^{-05}	1.52e^{-05}	1.49e^{-05}	1.00e^{+00}	1.49e^{-05}	1.53e^{-05}		1.01e^{-02}
**Mms7-MIC D7AD8A**	3.04e^{-03}	1.49e^{-05}	2.58e^{-04}	1.49e^{-05}	6.53e^{-01}	1.49e^{-05}	5.21e^{-02}	1.85e^{-05}	6.29e^{-01}	1.01e^{-02}	

Supplementary Table 3. G factor values from ESR experiments.

Sample	G factor	G factor (Low mT) [a]	G factor (High mT)
Control	2.131	2.419	1.839
MamC-MIC	2.066	2.337	1.851
MamC-MIC D14A	2.027	2.225	1.883
Mms6-MIC	2.063	2.326	1.869
Mms6-MIC D12A	2.023	2.232	1.876
Mms6-MIC E13A	2.052	2.329	1.872
Mms6-MIC D12AE13A	2.027	2.243	1.883
Mms7-MIC	2.099	2.441	1.832
Mms7-MIC D7A	2.183	2.509	1.847
Mms7-MIC D8A	2.031	2.261	1.883
Mms7-MIC D7AD8A	2.049	2.321	1.858

[a] mT is corresponds to the typical ESR field B_0.
